# Effect of interpregnancy weight change on perinatal outcomes: systematic review and meta-analysis

**DOI:** 10.1186/s12884-019-2566-2

**Published:** 2019-10-28

**Authors:** Noor E. W. D. Teulings, Katya L. Masconi, Susan E. Ozanne, Catherine E. Aiken, Angela M. Wood

**Affiliations:** 1 Department of Public Health and Primary Care, University of Cambridge, 2 Worth’s Causeway Cambridge, Cambridge, CB1 8RN UK; 2 University of Cambridge Metabolic Research Laboratories and MRC Metabolic Diseases Unit, Wellcome Trust-MRC Institute of Metabolic Science, Addenbrooke’s Hospital, Cambridge, CB2 0QQ UK; 3 Department of Obstetrics and Gynaecology, University of Cambridge, Box 223, The Rosie Hospital and NIHR Cambridge Comprehensive Biomedical Research Centre, Cambridge, UK

**Keywords:** BMI, Hypertensive disorders of pregnancy, Interpregnancy weight change, Meta-analysis, Obesity, Perinatal complications, Systematic review

## Abstract

**Background:**

Although obesity is a well-known risk factor for adverse pregnancy outcomes, evidence is sparse about the effects of interpregnancy weight change on the risk of adverse perinatal complications in a subsequent pregnancy. The current study aims to assess the effect of interpregnancy weight change on the risk of developing gestational diabetes, pre-eclampsia, pregnancy induced hypertension, preterm birth, or delivering a large- or small-for-gestational age neonate.

**Methods:**

Pubmed, Ovid Embase, ClinicalTrial.gov and the Cochrane library were systematically searched up until July 24th, 2019. Interpregnancy weight change was defined as the difference between pre-pregnancy weight of an index pregnancy and a consecutive pregnancy. Inclusion criteria included full text original articles reporting quantitative data about interpregnancy weight change in multiparous women with any time interval between consecutive births and the risk of any perinatal complication of interest. Studies reporting adjusted odds ratios and a reference group of − 1 to + 1 BMI unit change between pregnancies were harmonised by meta-analysis.

**Results:**

Twenty-three cohort studies identified a total of 671,906 women with two or more consecutive pregnancies. Seven of these studies were included in the meta-analysis (280,672 women). Interpregnancy weight gain was consistently associated with a higher risk of gestational diabetes, pre-eclampsia, pregnancy induced hypertension and large-for-gestational age births. In contrast, interpregnancy weight loss was associated with a lower risk of delivering a large-for-gestational age neonate. The effect magnitude (relative risk) of interpregnancy weight gain on pregnancy induced hypertension or delivering a large-for-gestational age neonate was greater among women with a normal BMI in the index pregnancy compared to women with a starting BMI ≥25 kg/m^2^.

**Conclusion:**

These findings confirm that interpregnancy weight change impacts the risk of developing perinatal complications in a subsequent pregnancy. This provides evidence in support of guidelines encouraging women to achieve post-partum weight loss, as their risk of perinatal complications might be minimised if they return to their pre-pregnancy weight before conceiving again.

Prospectively registered with PROSPERO (CRD42017067326).

## Background

Obesity is an increasing global health concern, with more than 1.9 billion adults worldwide being overweight [1] and approximately one in two US women of childbearing age now being considered overweight or obese [2]. Considerable evidence exists showing serious perinatal complications associated with obesity in pregnancy including gestational diabetes (GDM), pre-eclampsia (PE) and neonatal death [3]. There is also an increased risk of complications such as fetal growth restriction and preterm birth amongst underweight women [4]. However, evidence is sparse about the effect of interpregnancy weight change on the risk of adverse outcomes in subsequent pregnancies. Current NICE guidelines in the UK recommend that overweight or obese women are referred for weight loss support at the 6–8 week postnatal check-up [5] despite limited evidence to support widespread implementation of such health promotion strategies and of benefit for future pregnancy outcomes [6].

The current study aimed to systematically synthesise the published evidence on the associations between interpregnancy weight change and common perinatal complications for both mother and child including GDM, PE, pregnancy induced hypertension (PIH), preterm birth (PTB), and delivery of a large- and small-for-gestational age neonate (LGA and SGA). Additionally, we compared the risk of these complications after interpregnancy weight change in women with a normal BMI and overweight or obese women, and where possible, we investigated the dose-response relationships.

## Methods

### Eligibility criteria, information sources, search strategy

The electronic databases PubMed, Ovid EMBASE, ClinicalTrials.gov and Cochrane Central were systematically searched until July 24th, 2019. The search strategy included terms relating to ‘interpregnancy’, ‘between pregnancy’, ‘weight change’ or ‘BMI’. These search terms were combined with the outcomes of interest (‘gestational diabetes’, ‘pre-eclampsia’, ‘pregnancy-induced hypertension’, ‘preterm birth’, ‘small-for-gestational age’ and ‘large-for-gestational age’) and synonyms of these outcomes (for full search string see Additional file 3: Table S1). Furthermore, we cross-referenced selected papers for additional articles to include. The studies identified were uploaded onto Covidence, an online tool for screening of papers for systematic reviews (www.covidence.org). The review protocol was designed a priori and registered with PROSPERO under registration number CRD42017067326.

### Study selection

Studies were selected using the following predetermined inclusion criteria: [i] interpregnancy weight change reported in kilogram (kg), BMI units (kg/m^2^) or percentage body weight change in multiparous women with any time interval between the consecutive births, [ii] any of the perinatal outcomes of interest in the subsequent pregnancy, and [iii] observational, cohort or case-controlled human study design with a sample size ≥50, that were reported in English. When studies reported data from overlapping study populations, the study with the largest sample size was selected for inclusion. Information extracted from each study included country of research, study cohort name (if applicable), study period, sample size, study inclusion criteria, methods of weight reporting, definition of reference group, diagnostic criteria for perinatal outcomes and demographics that studies adjusted for. All study selection, full text screening, and data extraction was undertaken independently by two researchers (NEWDT and KLM), following PRISMA guidelines [7]. Disagreements were decided through a third opinion (AMW).

### Data synthesis

Interpregnancy weight change was defined as the difference between pre-pregnancy weight in the index pregnancy, defined as the earliest recorded pregnancy, and pre-pregnancy weight in the subsequent pregnancy. Interpregnancy weight gain and loss were defined on two categorical scales; (i) for the meta-analysis we utilised categories of > 1 BMI unit interpregnancy weight loss, BMI gain between 1 and 2 units, BMI gain between 2 and 3 units or BMI gain of more than 3 units and (ii) for the dose-response analysis we utilised a BMI change of 0, 1, 2 or 3+ units. Crude odds ratios (calculated from studies providing relevant counts) and adjusted odds ratios for each outcome of interest were extracted from the selected publications.

Random effects meta-analysis was used to synthesize the odds ratios for weight change categories. To ensure a consistent reference group, only studies that employed a reference group of interpregnancy weight change between 1-unit weight loss and 1-unit weight gain were included. Heterogeneity was assessed using the I^2^ statistic.

We conducted a separate analysis comparing interpregnancy weight change and the risk of developing adverse perinatal outcomes in women with a normal BMI (< 25 kg/m^2^) versus women with an overweight BMI (≥25 kg/m2), at the start of their index pregnancy. To do so, adjusted odds ratios for both BMI categories were extracted from the publications and summarised by random effects meta-analysis.

Dose-response relationships were assessed by plotting association measurements from studies providing multiple weight gain categories. Where ranges of BMI changes were reported, the midpoint of the category was used (e.g. 1.5 BMI units change for the category weight change between 1 and 2 BMI units).

Statistical analysis and graphical presentation were performed using the *metafor* package in R for Windows, version 3.4.2.

### Assessment of risk of bias

A sensitivity analysis was undertaken to assess potential impact of bias in individual studies by excluding studies that scored below 5 out of 9 points in the Newcastle-Ottawa Scale (NOS [8]) quality scoring assessment (Additional file 4: Table S2). Furthermore, leave-one-study-out analyses were conducted to identify whether one study leveraged the overall effect size estimate.

## Results

### Study selection

**Fig. 1:**
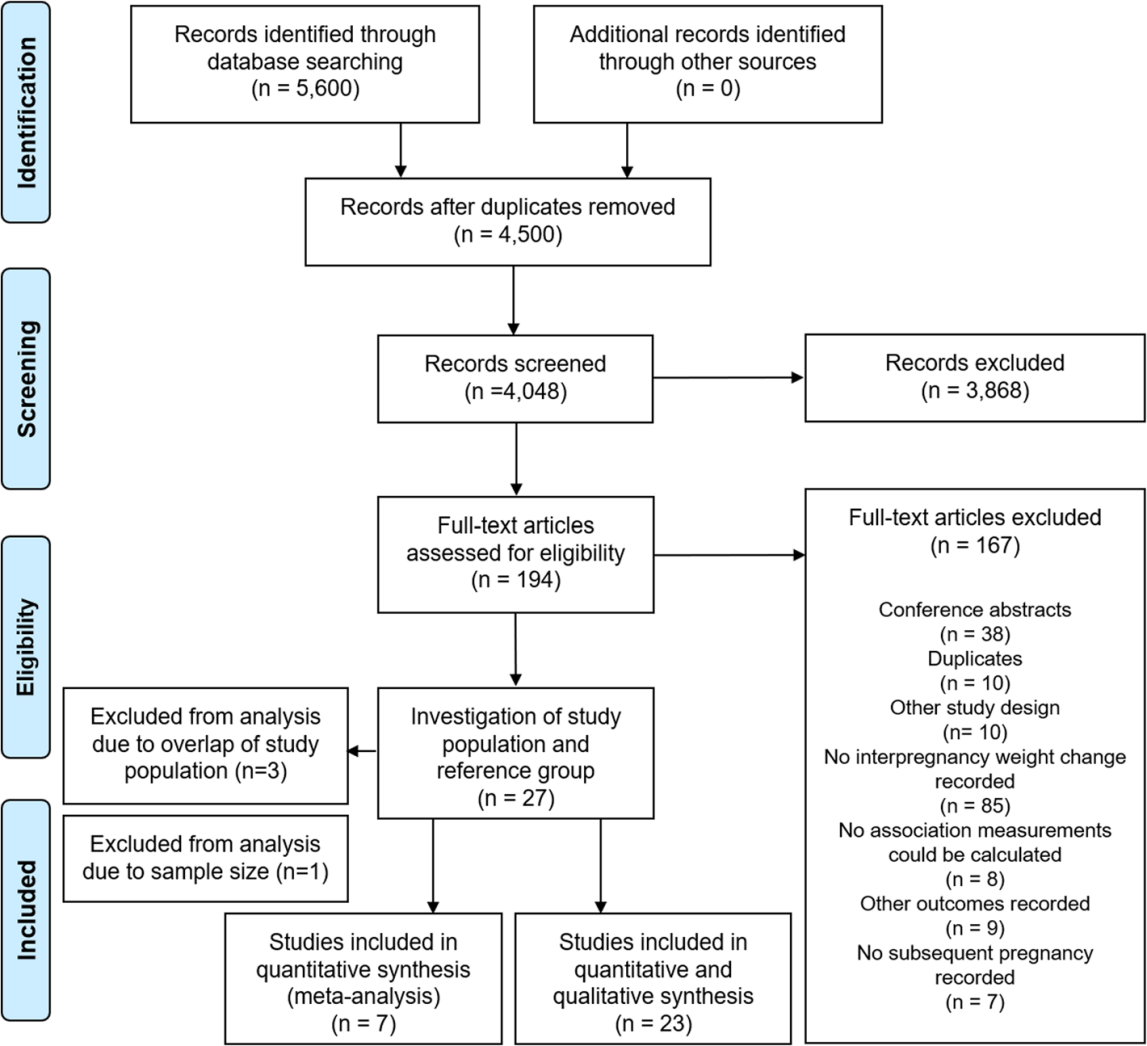
Flow diagram of study inclusion and exclusion

We identified and screened 4500 unique publications and included 194 articles for full text review (Fig. 1). A total of 27 studies were eligible for inclusion. Three studies were excluded due to overlapping study populations [9–11] and one was excluded because of a sample size < 50 women [12]. From the remaining 23 studies selected to take forward, a total of 671,906 women were identified for inclusion in the review (Table 1). Eighteen studies included only nulliparous women at the index pregnancy. The proportion of women older than 35 years varied between studies from 3 to 33%. All studies were conducted in Western populations, although this was not an inclusion criterion. Seven studies, comprising of 280,672 women, were included in the meta-analysis.

**Table 1:** Characteristics of studies investigating the association between interpregnancy weight change and adverse perinatal outcomes

Author & publication date	Included in meta-analysis?^a^	Country	Study cohort (if applicable)	Study period	Sample size	Inclusion criteria	Reported weight	Reference group	Diagnostic criteria	Confounders adjusted for
Bogaerts et al. 2013 [25]	Yes	Belgium	Study Centre for Perinatal Epidemiology database	2009–2011	7897	First two consecutive births	Self-reported weight and height	±1 BMI unit	GDM: not clarified PIH: not clarified	Prepregnancy BMI at first pregnancy, interpregnancy interval, gestational age at first delivery, maternal age, gestational weight gain, complications at first pregnancy (GDM, PIH, induction of labour, CS, malformations and mortality)
Bender et al. 2018 [46]	No	USA	Hospital of Pennsylvania retrospective cohort	2005–2010	537	Singleton livebirth followed by consecutive pregnancy	Weight measured at first antenatal visit, self-reported height	Stable BMI category	GDM: Carpenter–Coustan criteria for the 3-h glucose tolerance test PIH: Task Force on Hypertension in Pregnancy PTB: < 37 weeks	Maternal age, GDM in prior pregnancy, prepregnancy BMI category
Benjamin et al. 2019 [39]	No	USA	Texas linked siblings pair	2005–2012	2481	Birth certificates linked with older live birth, singleton sibling	Self-reported weight and height	0 to < 1 BMI units weight gain	SGA: Not reported LGA: Not reported PTB: Not reported	Prepregnancy BMI at sibling pregnancy, ethnicity, smoking status, gestational weight gain, height, maternal age and education
Chen et al. 2009 [26]	No	USA	Collaborative Perinatal Project	1959–1966	1892	Singleton livebirth followed by consecutive singleton pregnancy	Self-reported weight and height	− 0.32 to 1.48 BMI units	PTB: < 37 weeks	Maternal age, research centre, race, smoking status, socio-economic index, marital status and interpregnancy interval
Cheng et al. 2003 [27]	No	USA	Missouri maternally linked cohort	1989–1997	14,114	Second-born SGA infants	Self-reported weight and height	No change in BMI	SGA: <10th percentile	Not reported
Crosby et al. 2017 [47]	No	Ireland	Follow up of ROLO study	2007–2015	280	Secundigravida who previously gave birth to macrosomic (> 4.0 kg) baby	Weight and height measured at first antenatal visit	No interpregnancy weight gain (not further specified)	GDM: Not specified	No adjusted model available
Ehrlich et al. 2011 [24]	Yes	USA	Kaiser Permanente Northern California	1996–2006	22,351	Women without recognised diabetes before pregnancy, first and second live born singletons	Measured by clinician at time of alpha fetoprotein test (mean GA 16.9 weeks)	± 1.0 BMI unit	GDM: According ADA criteria	Maternal age, race, ethnicity, place of birth, GDM status in first pregnancy, prepregnancy BMI in first pregnancy, gestational age, interpregnancy interval
Getahun et al. 2007 [28]	No	USA	Missouri vital record system	1989–1997	136,884	No history of pre-eclampsia in index pregnancy, delivering second baby.	Self-reported weight and height	Normal BMI (18.5–24.9 kg/m^2^) in both pregnancies	PE: hypertension and proteinuria beyond 20th week gestation in women normotensive before pregnancy	Maternal age, race, education, marital status, prenatal care, smoking status and interpregnancy interval
Getahun et al. 2007 [29]	No	USA	Missouri vital record system	1989–1997	146,227	First two consecutive singleton pregnancies	Self-reported weight and height	Normal BMI (18.5–24.9 kg/m^2^) in both pregnancies	LGA: ≥90th percentile	Maternal age, race, education, marital status, prenatal care, smoking status, alcohol during pregnancy, marital status and interpregnancy interval
Glazer et al. 2004 [36]	No	USA	Washington State Longitudinal Births Database	1992–1998	4102	Non-diabetic women with weight ≥ 200lbs. with ≥2 singleton births.	Pre-pregnancy weight from birth certificate, unspecified how measured	± 10 lb	GDM: not clarified	Maternal age, gestational weight gain in index pregnancy and gestational weight gain during subsequent pregnancy
Hoff et al. 2009 [30]	No	USA	Missouri birth certificates	1995–2004	1035	First two consecutive singleton pregnancies in overweight women	Pre-pregnancy weight from birth certificate, unspecified how measured	overweight BMI (25.0–29.9 kg/m^2^) in both pregnancies	PIH: not clarified PTB: < 37 weeks	No adjusted model available
Jain et al. 2013 [31]	No	USA	Missouri vital record system	1998–2005	10,444	First two consecutive singleton pregnancies with a BMI ≥ 30 at index pregnancy.	Self-reported weight and height	± 2 BMI units	SGA: <10th percentile LGA:< 90th percentile	Maternal ae, race, martial status, education, socioeconomic status, obesity status in first pregnancy, gestational weight gain, smoking, PE, prenatal care, previous SGA or LGA birth, DM, hypertension, renal or cardiac disease
Knight-Agarwal et al. 2016 [32]	Yes	Australia	Birthing Outcome System	2008–2013	14,875	Women of all parity with subsequent pregnancies.	Weight and height recorded at first antenatal visit (mean GA not reported)	± 1 BMI unit	GDM: not clarified	Maternal age, parity, country of birth, smoking status
Kruse et al. 2015 [33]	No	Denmark	–	2009–2013	72	Primiparas with a history of GDM	Unspecified how weight was recorded	No change in BMI units	GDM: ≥9.0 mmol/L blood glucose 2 h after OGTT.	No adjusted model available
Lynes et al. 2017 [22]	Yes	USA	NICHD Consecutive Pregnancy Study	2002–2010	46,521	First two consecutive singleton births	Unspecified how weight was recorded	± 1 BMI unit	GDM: not clarified PIH: ≥140 mmHg systolic and ≥ 90 mmHg diastolic without proteinuria PE: ≥140 mmHg systolic and ≥ 90 mmHg diastolic with proteinuria	Maternal race, interpregnancy interval, maternal age, marital status, smoking status, alcohol use during second pregnancy, prepregnancy BMI, complication in first pregnancy (GDM, PE, PIH)
McBain et al. 2016 [34]	No	Australia	Women’s and Childeren’s Health Network	2000–2012	5371	First and second consecutive deliveries.	BMI units recorded at first antenatal visit (before GA 15 weeks)	±2 BMI units	GDM: not clarified PTB: not clarified SGA: <10th centile LGA: ≥90th centile	Maternal age, socioeconomic status, prepregnancy BMI in first pregnancy, smoking status, race, interpregnancy interval, first pregnancy outcome (GDM, PIH, birth method, LGA and SGA)
Pole et al. 1999 [37]	No	Canada	Nova Scotia Atlee Perinatal Database	1988–1996	19,932	Two or more singletons	Not stated	± 3% weight	GDM: two abnormal glucose values on a GTT according to Joslin Clinic or O’Sullivan criteria. PIH: BP ≥90 mmHg diastolic, twice in 24 h	Prepregnancy weight (in lbs) of index pregnancy, gestational age, marital status, previous CS, maternal age, gestational weight gain, GDM in previous pregnancy
Simonsen et al. 2013 [38]	No	USA	Maternally linked Utah birth and fetal records	1989–2007	8468	First three singleton live births.	Pre-pregnancy BMI from birth certificate (mean GA not reported)	BMI category unchanged	PTB: ≥20 and < 37 weeks	Maternal age, ethnicity, gestational weight gain, father on birth record, interpregnancy interval, subtype of previous PTB, gestational age at previous PTB, fetal death or anomaly in history
Sorbye et al. 2017 [23]	Yes	Norway	Medical Birth Registry of Norway	2006–2014	24,198	First and second delivery without GDM in index pregnancy	Unspecified how weight was recorded	± 1 BMI units	GDM: fasting glucose < 7.0 mmol/l and serum glucose after OGTT ≥7.8 mmol/l	Maternal age, country of birth, maternal education, smoking status, interpregnancy interval and year of delivery
Villamor et al. 2006 [20]	Yes	Sweden	Swedish Birth Register	1992–2001	151,025	First and second consecutive singleton births.	BMI units recorded at first antenatal visit (mean GA not reported)	± 1 BMI units	GDM: ICD-9648 W, ICD-10 O244. PE: ICD-9642E-642H, ICD-10 O11 and O14. PIH: ICD-9642D and 642X, ICD-10 O13 LGA: ≥2 SD above mean birthweight	Prepregnancy BMI in first pregnancy, height, interpregnancy interval, maternal age, country of birth, education, year of delivery, smoking status
Wallace et al. 2014 [21]	Yes	Scotland	Aberdeen Maternity and Neonatal Databank	1986–2007	12,740	First two consecutive births.	Weight and height recorded at first antenatal visit (mean GA not reported)	± 1 BMI units	PE: ISSHP definition PIH: ISSHP definition PTB: < 37 weeks SGA: <10th percentile LGA: ≥90th percentile	Prepregnancy BMI in first pregnancy, height inter-delivery interval, maternal age, year of delivery, smoking status, gestational age and fetal gender at second pregnancy.
Wallace et al. 216 [35]	No	Scotland	Aberdeen Maternity and Neonatal Databank	1986–2013	24,520	First two consecutive births and the same perinatal complication in both pregnancies	Weight and height recorded at first antenatal visit (mean GA not reported)	± 2 BMI units	PTB: < 37 weeks PE: ISSHP definition PIH: ISSHP definition SGA: <10th percentile LGA: ≥90th percentile	Prepregnancy BMI in first pregnancy, year of delivery, height, inter-delivery interval, maternal age, smoking status, gestational age and fetal sex at first and second pregnancy
Ziauddeen et al. 2019 [19]	Yes	England	Birth registry at Southampton Hospital	2003–2017	15,940	First two consecutive singleton live-birth pregnancies	Weight recorded at first antenatal visit; height self-reported	± 1 BMI unit	LGA: >90th percentile for GA	Baseline BMI, maternal age, education level, infertility treatment, smoking status, employment status, GDM in current pregnancy and interpregnancy interval

^a^To ensure a consistent reference group, only studies that employed a reference group of interpregnancy weight change between 1-unit weight loss and 1-unit weight gain were included. *GDM* gestational diabetes, *PE* pre-eclampsia, *PIH* pregnancy induced hypertension, *PTB* preterm birth, *SGA* small-for-gestational age, *LGA* large-for gestational age, *BMI* body mass index, *GA* gestational age, *CS* caesarean section, *DM* diabetes mellitus

### Synthesis of results

**Fig. 2:**
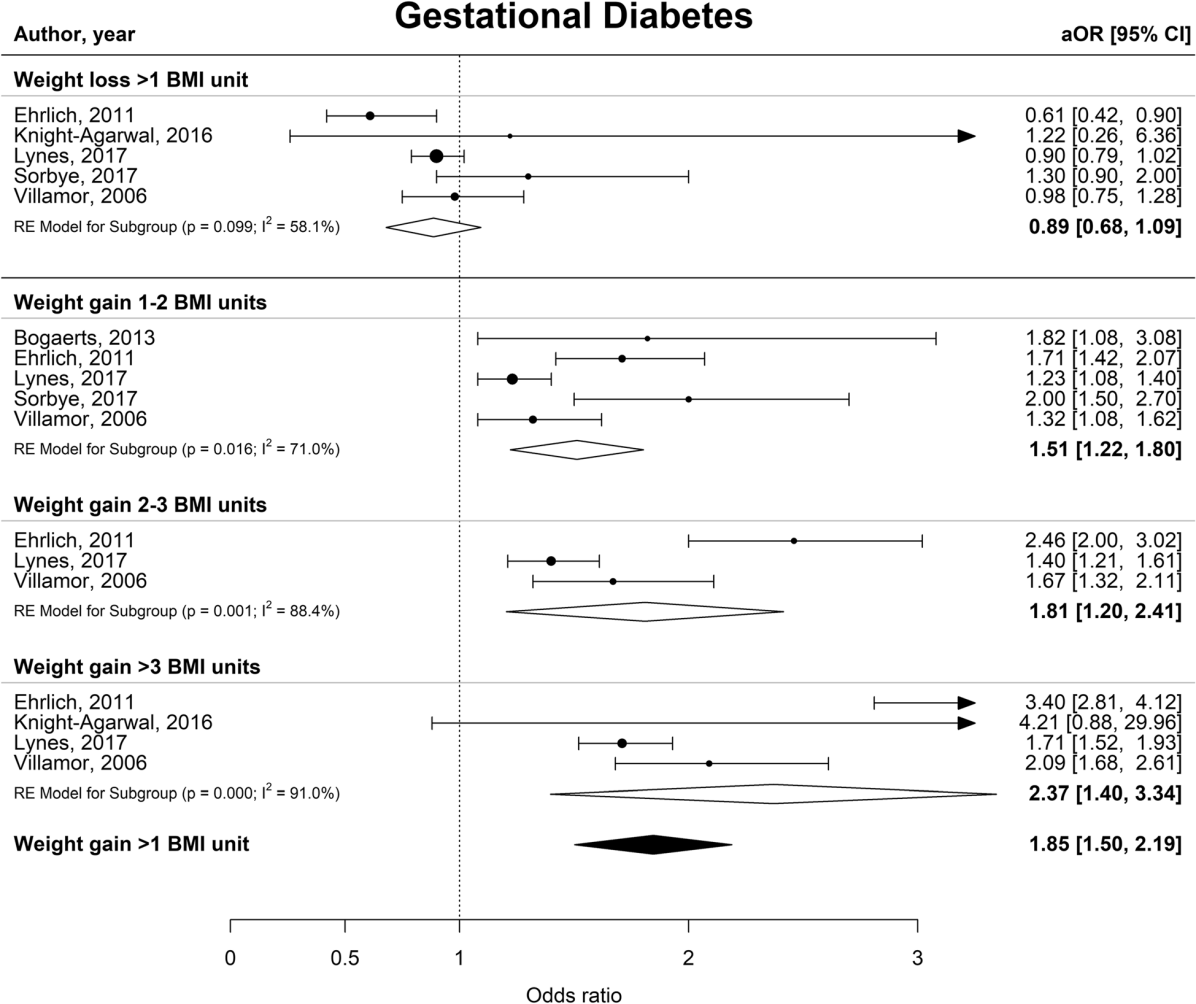
Forest plot from random effects meta-analysis showing the association between interpregnancy weight change and the risk of developing gestational diabetes in subsequent pregnancy. All adjusted odds ratios are relative to the reference category of interpregnancy weight change between − 1 and + 1 BMI units. BMI, body mass index (in kg/m^2^); aOR, adjusted odds ratio; CI, confidence interval

**Fig. 3:**
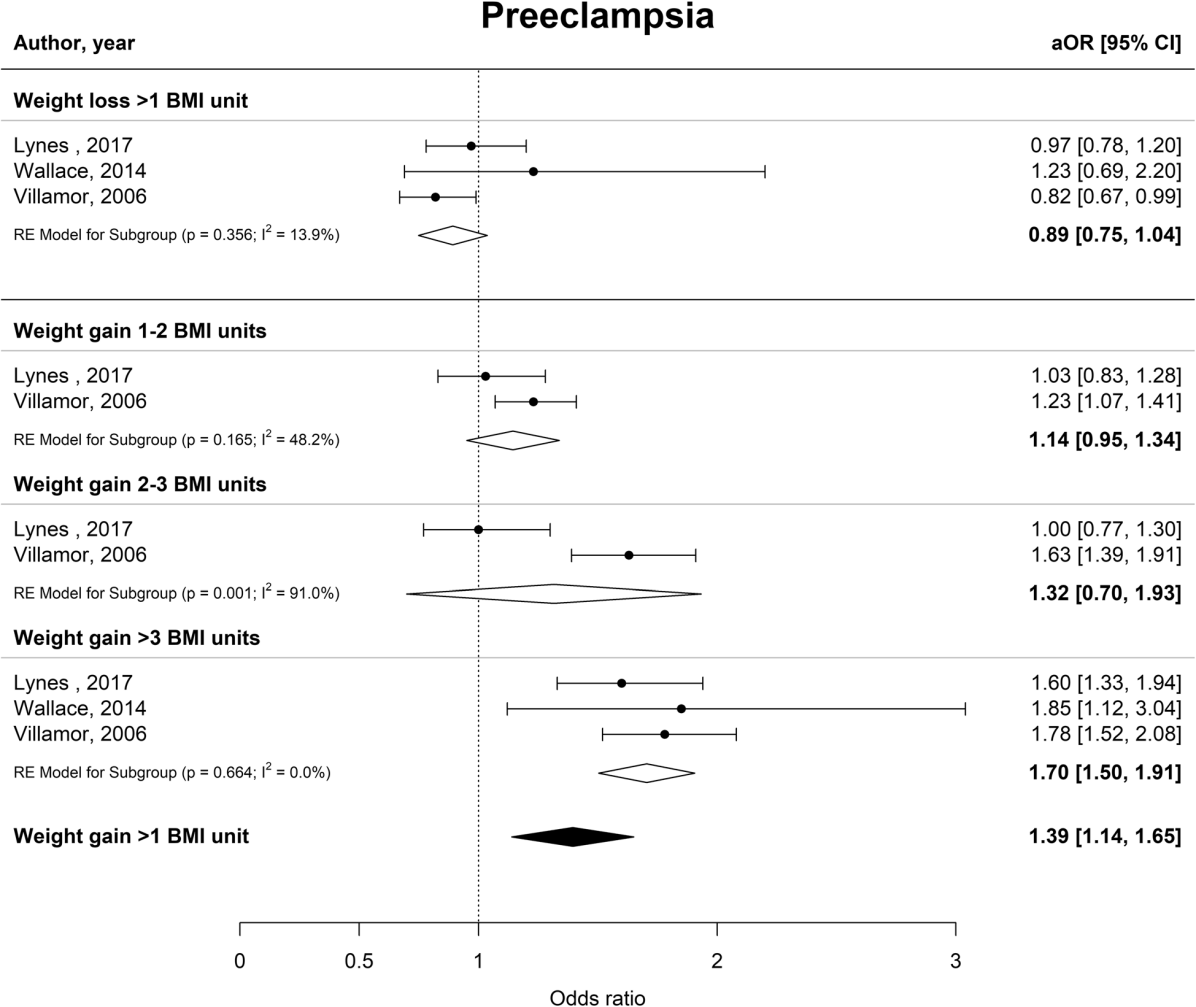
Forest plot from random effects meta-analysis showing the association between interpregnancy weight change and the risk of developing pre-eclampsia in subsequent pregnancy. All adjusted odds ratios are relative to the reference category of interpregnancy weight change between − 1 and + 1 BMI units. BMI, body mass index (in kg/m^2^); aOR, adjusted odds ratio; CI, confidence interval

**Fig. 4:**
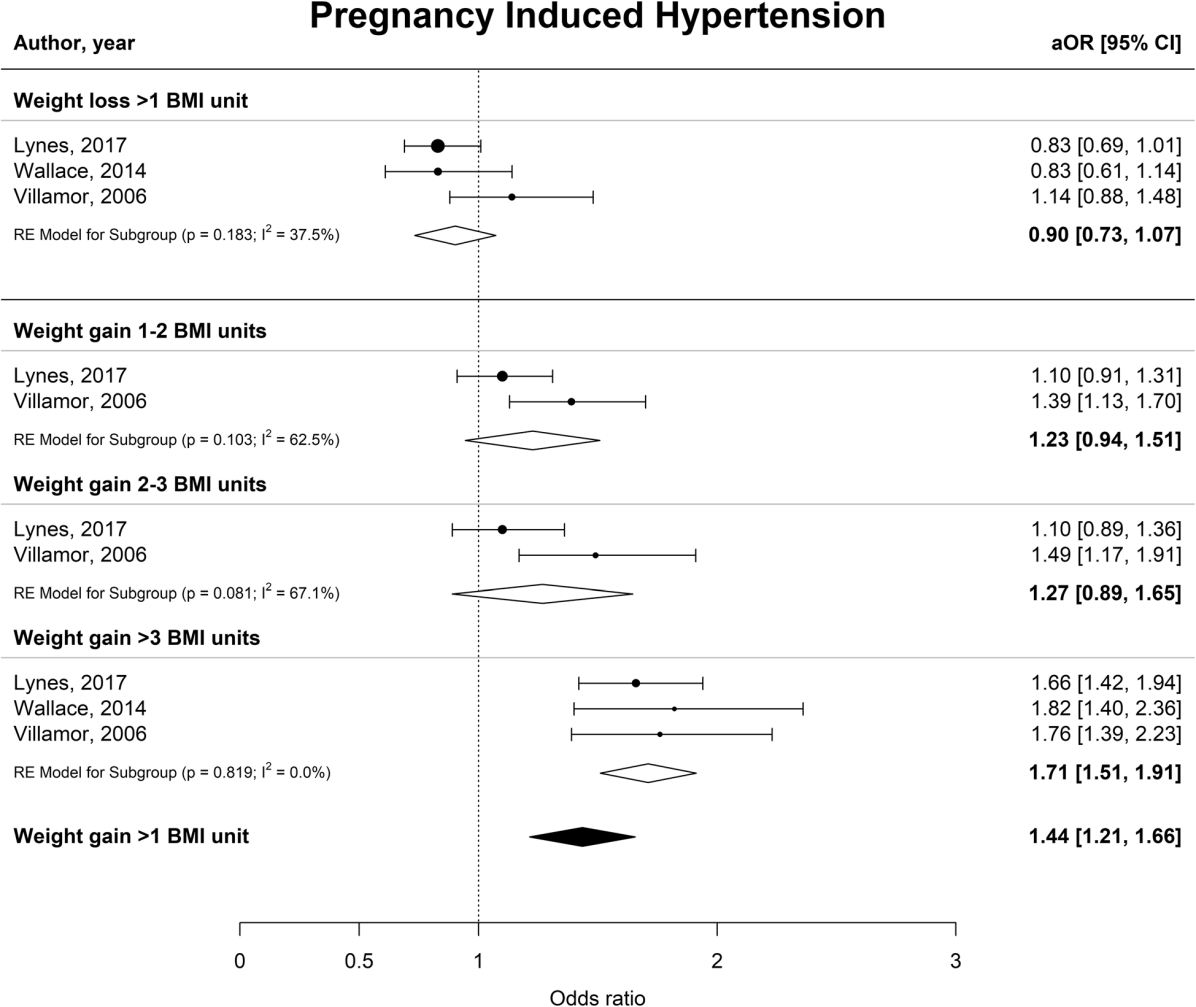
Forest plot from random effects meta-analysis showing the association between interpregnancy weight change and the risk of developing pregnancy induced hypertension in subsequent pregnancy. All adjusted odds ratios are relative to the reference category of interpregnancy weight change between − 1 and + 1 BMI units. BMI, body mass index (in kg/m^2^); aOR, adjusted odds ratio; CI, confidence interval

**Fig. 5:**
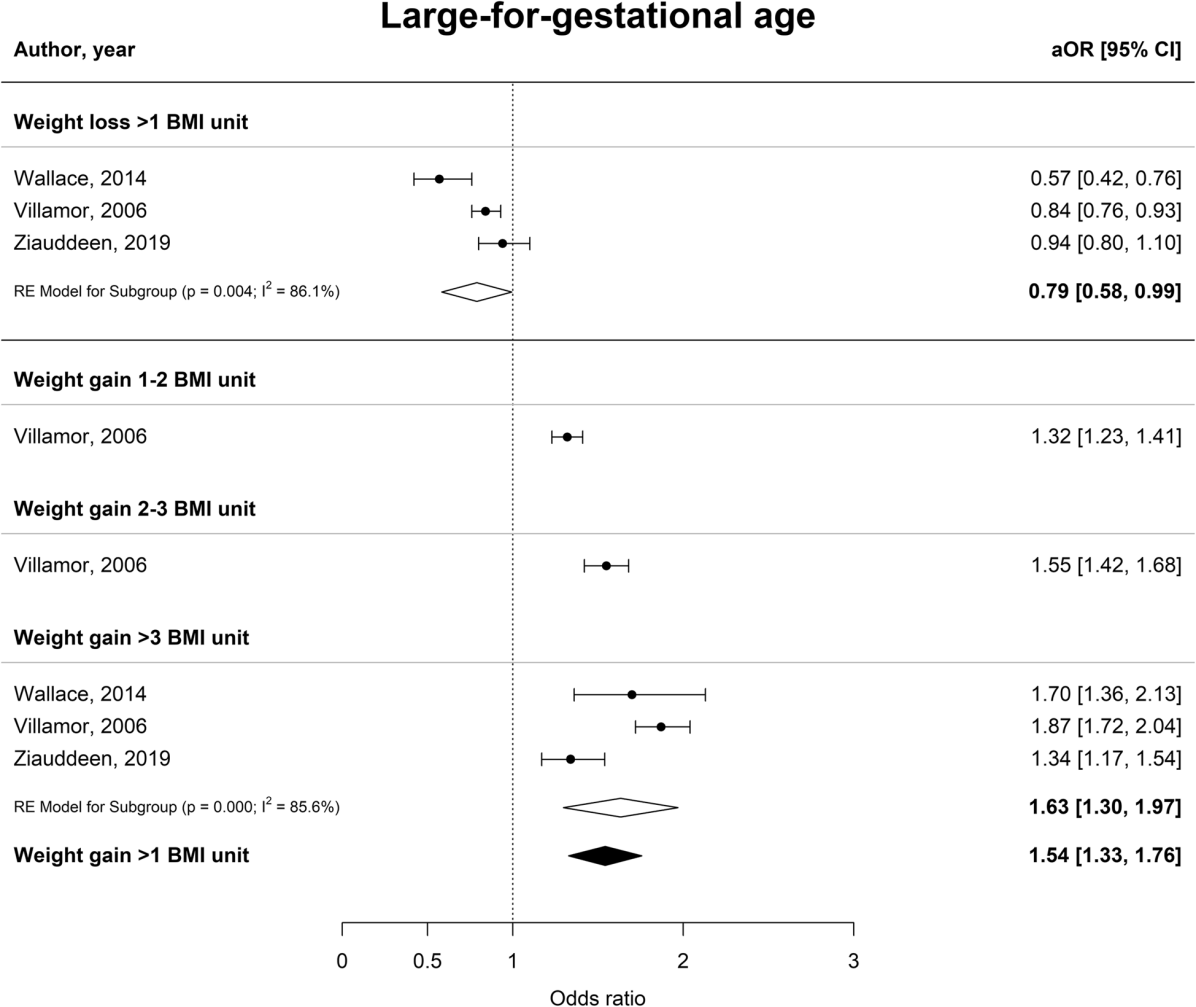
Forest plot from random effects meta-analysis showing the association between interpregnancy weight change and the risk of delivering a large-for-gestational age neonate in subsequent pregnancy. All adjusted odds ratios are relative to the reference category of interpregnancy weight change between − 1 and + 1 BMI units. BMI, body mass index (in kg/m^2^); aOR, adjusted odds ratio; CI, confidence interval

Interpregnancy weight gain of between 1 and 2 BMI units was associated with a 51% higher risk of developing GDM (aOR 1.51 [1.22–1.80], I^2^ = 70.1%), whereas an increase of 2–3 or more than 3 BMI units was associated with an 81 and 137% higher risk (aOR 1.81 [1.20–2.41], I^2^ = 88.4% and aOR 2.37 [1.50–3.34], I^2^ = 91.0% respectively) (Fig. 2). Furthermore, interpregnancy weight gain of more than 3 BMI units was associated with a higher risk of PE or PIH (aOR 1.70 [1.50–1.91], I^2^ = 0.0% and aOR 1.71 [1.51–1.91] I^2^ = 0.0% respectively) (Figs. 3 and 4). The association between interpregnancy weight change and the risk of delivering an LGA neonate could only be estimated for a weight gain > 3 BMI units, and showed a 63% higher risk (aOR 1.63 [1.30–1.97], I^2^ = 85.6%) (Fig. 5). In contrast, interpregnancy weight loss of > 1 BMI unit was associated with a lower risk of delivering an LGA neonate, (aOR 0.79 [0.58–0.99], I^2^ = 86.1%) (Fig. 5), but there was no conclusive evidence of association of interpregnancy weight loss with the risk of developing GDM, PE or PIH (Figs. 2, 3 and 4). There was an insufficient number of studies to conduct a meta-analysis on adjusted odds ratios for the outcomes of SGA and PTB. A meta-analysis combining the crude odds ratios rather than adjusted ratios showed a significant higher risk of developing PE (cOR 1.31 [1.09–1.53], I^2^ = 75.1%), but showed similar results for the association between interpregnancy weight gain and the risk of developing GDM, PE or PIH (Additional file 1: Figure S1 for interpregnancy weight loss and Additional file 2: Figure S2 for weight gain). For the outcomes of SGA and PTB, meta-analyses of crude odds ratios showed interpregnancy weight loss of > 1 BMI unit was associated with a higher risk of delivering a SGA neonate or delivering preterm (cOR 1.53 [1.35–1.71], I^2^ = 0.0% and cOR 1.45 [1.21–1.69], I^2^ = 26.7%] respectively), but there was no evidence of association with interpregnancy weight gain (Additional file 1: Figure S1 and Additional file 2: Figure S2).

**Fig. 6:**
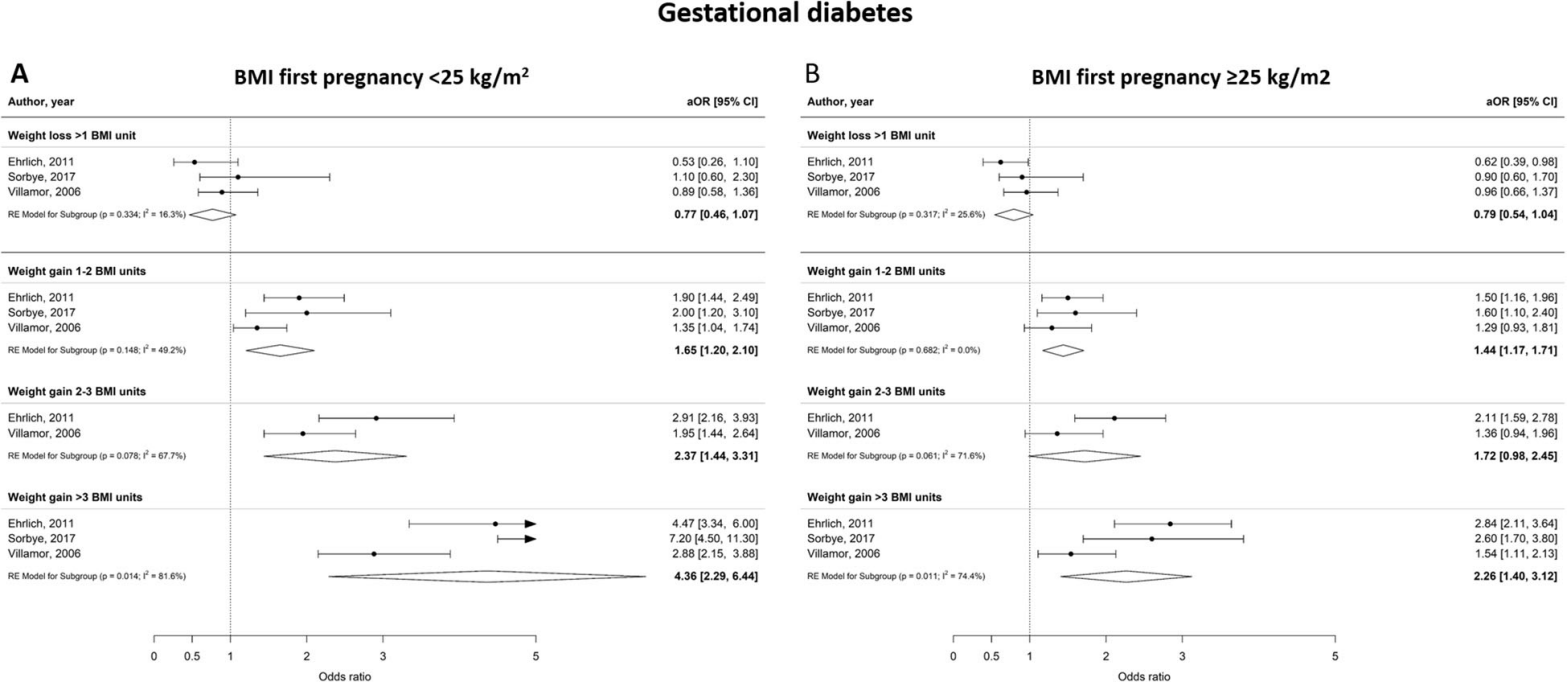
Forest plot from random effects meta-analysis showing association between interpregnancy weight change and the risk of developing gestational diabetes, stratified by BMI category at the start of index pregnancy. **a** Normal weight classified as BMI < 25 kg/m^2^; **b** Overweight classified as BMI ≥25 kg/m^2^. All adjusted odds ratios are relative to the reference category of interpregnancy weight change between − 1 and + 1 BMI units. BMI, body mass index (in kg/m^2^); aOR, adjusted odds ratio; CI, confidence interval

**Fig. 7:**
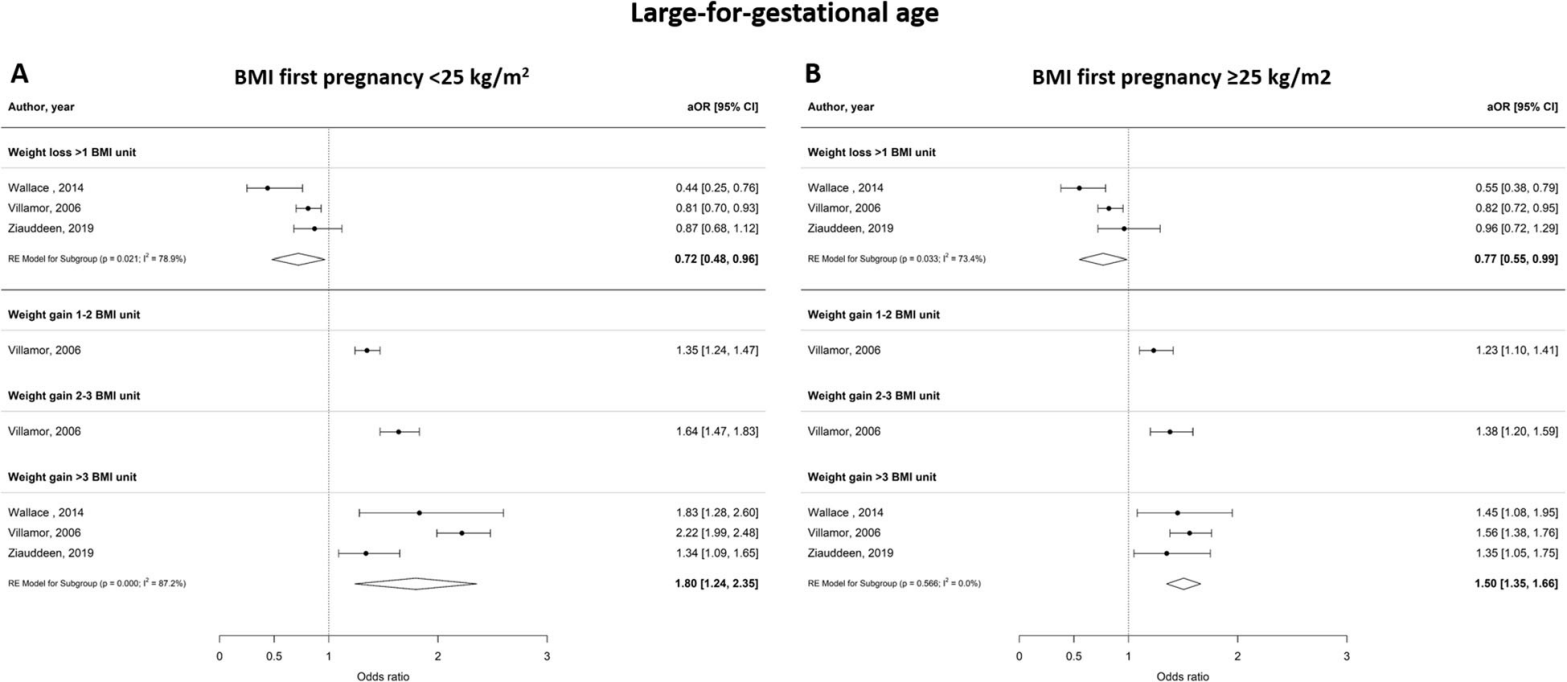
Forest plot from random effects meta-analysis showing association between interpregnancy weight change and the risk of delivering a large-for-gestational age neonate, stratified by BMI category at the start of index pregnancy. **a** Normal weight classified as BMI < 25 kg/m^2^; **b** Overweight classified as BMI ≥25 kg/m^2^. All adjusted odds ratios are relative to the reference category of interpregnancy weight change between − 1 and + 1 BMI units. BMI, body mass index (in kg/m^2^); aOR, adjusted odds ratio; CI, confidence interval

**Fig. 8:**
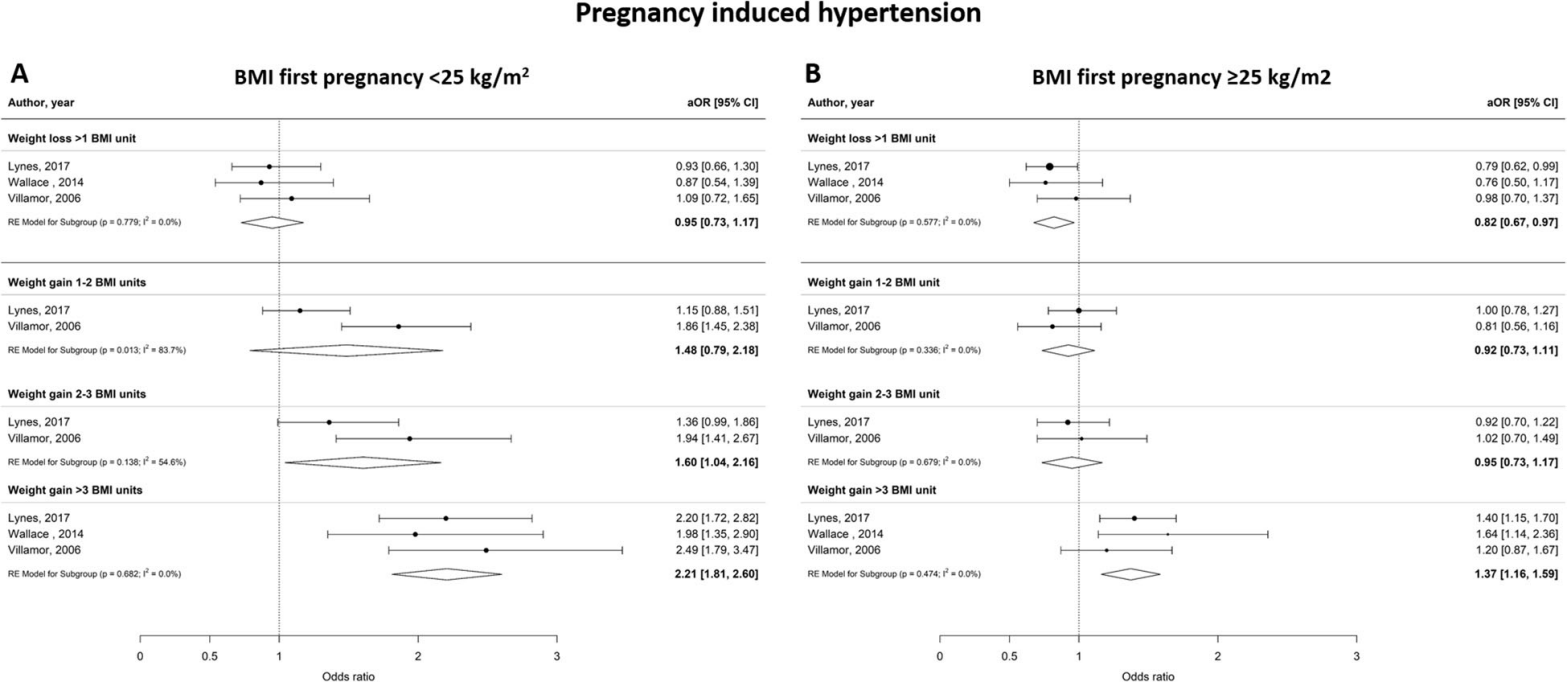
Forest plot from random effects meta-analysis showing association between interpregnancy weight change and the risk of developing pregnancy induced hypertension, stratified by BMI category at the start of index pregnancy. **a** Normal weight classified as BMI < 25 kg/m^2^; **b** Overweight classified as BMI ≥25 kg/m^2^. All adjusted odds ratios are relative to the reference category of interpregnancy weight change between − 1 and + 1 BMI units. BMI, body mass index (in kg/m^2^); aOR, adjusted odds ratio; CI, confidence interval

Figures 6, 7 and 8 show the odds ratios for the risk of developing an adverse perinatal outcome after interpregnancy weight gain, stratified by BMI category in the index pregnancy (normal weight; BMI < 25 kg/m^2^ versus overweight; BMI ≥25 kg/m^2^). Women with a normal weight at the start of the index pregnancy had a higher risk of developing GDM after interpregnancy weight gain > 3 BMI units (aOR 4.36 [2.29–6.44], I^2^ = 81.6%) compared to women with an overweight BMI (aOR 2.26 [1.40–3.12], I^2^ = 74.4%) (Fig. 6a and b). Similarly, women with a BMI < 25 kg/m^2^ were at higher risk of delivering a LGA neonate after interpregnancy weight gain > 3 BMI units than women with BMI ≥25 kg/m^2^ (aOR 1.80 [1.24–2.35], I^2^ = 87.2% versus aOR 1.50 [1.35–1.66], I^2^ = 0.0% respectively) (Fig. 7a and b). Women with a normal BMI at the start of their index pregnancy were at higher risk of developing PIH in a subsequent pregnancy after interpregnancy weight gain of 2–3 BMI (aOR 1.60 [1.04–2.16, I^2^ = 54.6%) and > 3 BMI units (aOR 2.21 [1.81–2.60], I^2^ = 0.0%), compared to women with an overweight BMI (2–3 units gain; aOR 0.95 [0.73–1.17], I^2^ = 0.0%, > 3 units gain; aOR 1.37 [1.16–1.59], I^2^ = 0.0%) (Fig. 8a and b). We did not find differential effects of interpregnancy weight loss between women with a normal BMI and women with an overweight BMI on the risk of developing GDM, PIH or delivering an LGA neonate.

**Fig. 9:**
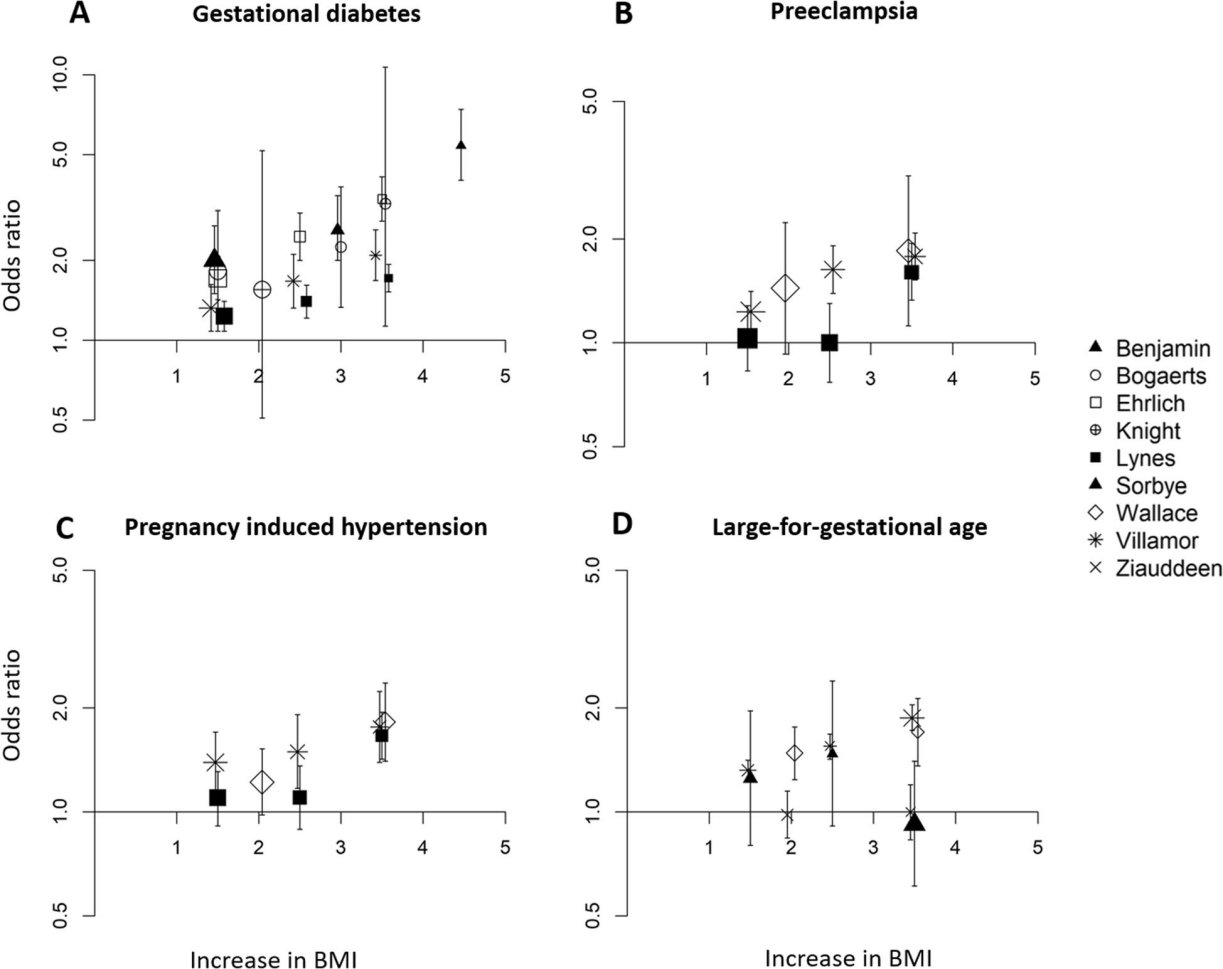
Dose-response curve with line of best fit for the increase in odds ratio of developing perinatal complications after interpregnancy weight gain. **a** Gestational Diabetes. **b** Pre-eclampsia. **c** Pregnancy Induced Hypertension **d** Large-for-gestational age. Where ranges of BMI changes were reported, the midpoint category was utilised (e.g. 1.5 BMI units change for the category weight change between 1 and 2 BMI units). aOR, adjusted odds ratio. BMI, body mass index (in kg/m^2^)

There was an approximate log-linear association between interpregnancy weight gain and the risk of developing GDM (Fig. 9a), PE (Fig. 9b) or PIH (Fig. 9c) and delivering a LGA neonate (Fig. 9d).

### Risk of bias of included studies

After assessing the study selection criteria, comparability of cases and controls and outcome assessments through the NOS, we identified four studies of poor quality (NOS score < 5, Additional file 4: Table S2). However, as these studies did not employ a reference group of ±1 BMI unit, they were already excluded from the meta-analyses. Leave-one-out-analyses showed that removing the study by Villamor et al. made the association between GDM and interpregnancy weight change of 2–3 or > 3 BMI units not significant. We did not find evidence that the outcomes for PE or PIH were driven by one study. For the outcome of delivering an LGA neonate, leave-one-out analyses could not be conducted due to only two studies being included in the meta-analysis.

## Discussion

### Main findings

This study systematically summarises and examines the published literature on the associations between interpregnancy weight change and several common perinatal outcomes. Our main findings are that interpregnancy weight gain is associated with a higher risk of developing GDM, PE, PIH and delivering an LGA neonate, while interpregnancy weight loss is associated with a lower risk of delivering an LGA neonate. BMI at the start of the index pregnancy possibly modifies the risk of developing GDM, PIH or delivering an LGA neonate after interpregnancy weight gain. Furthermore, we identify a positive approximately log-linear relationship between interpregnancy weight gain and the risk of developing GDM, PE, PIH or delivering a LGA neonate.

### Comparison with existing literature

Our study confirms the associations between interpregnancy weight gain and the risk of developing GDM and LGA, as also shown in a recent meta-analysis [13]. Our research additionally summarises the effect of interpregnancy weight change on the risk of developing hypertensive disorders in pregnancy. However, our meta-analysis is to the authors knowledge the first study to show that gaining weight between pregnancies increases the risk of developing hypertensive disorders in the subsequent pregnancy. The observation that starting BMI possibly modifies this association is important for women with a healthy BMI at the start of their index pregnancy, as research often emphasises the risk associated with being overweight or obese, and women with a healthy BMI might not be aware of the risk that comes with (small) interpregnancy weight gain. Although the risks of (excessive) gestational weight gain [14] and high pre-pregnancy BMI [15, 16] on perinatal outcomes are well understood, the effects of interpregnancy weight gain are relatively unknown and are essential to understand in order to guide women in periconception and perinatal weight management.

Our study shows an approximate log-linear association between BMI gain and the risk of developing GDM or hypertensive disorders in pregnancy. This result contributes towards understanding the association between maternal weight and pregnancy complications. Linear dose-response associations are established between obesity and the incidence of GDM, PE and PIH [17], between adiposity and pre-eclampsia [18], as well as maternal weight and pre-eclampsia [15] and GDM [16]. Our identified associations emphasise the detrimental effects of (small amounts of) weight gain, additional to the influence of absolute BMI. This can contribute towards understanding the importance of postpartum weight management and highlights the need for the development of clinical guidelines.

### Strengths and limitations

A strength of our study is we ensured a homogenous reference group (i.e. a BMI change ≤1 kg/m^2^) for our meta-analysis rather than including studies with very different reference groups [13]. Furthermore, we only harmonised studies reporting adjusted odds ratios, which all considered maternal age, country of origin, social economic status and smoking status as potential confounders. Nevertheless, our study has several limitations. First, between-study heterogeneity remained, arising from differences in outcome definitions and demographics, such as parity and age, and potentially differences in length of interpregnancy intervals and prevalence of perinatal complications. Of the studies selected for meta-analysis, only Lynes et al. did not restrict to nulliparous women, although removing this study had little impact on the results. Second, GDM, PE and PIH were either not defined in publications or the definitions of these adverse outcomes differed between studies, hence caution is needed when comparing effect estimates between studies. Third, it was not possible to consistently assess the impact of previous pregnancy complications, which may lead to excessive interpregnancy weight changes and a higher risk of subsequent pregnancy complications. Fourth, studies varied in the way they measured pre-pregnancy weight, with the majority of studies using self-reported weight (and height) to calculate BMI and interpregnancy weight change. Although evidence suggests that maternal reports of pre-pregnancy weight are in general consistent with clinical records [42], bias due to systematic over- or underreporting cannot be excluded. We can also not exclude the possibility of publication bias, as this could not be assessed due to the small number of studies available per adverse outcome and funnel plot assessment is generally not recommended with less than 10 studies [43]. Lastly, we were unable to make the distinction between spontaneous preterm birth and medically induced preterm birth. We hypothesise that an increased risk of preterm birth is at least partly related to the increased risk of carrying an SGA neonate, as (suspected) growth restriction is one of the main causes of medically induced premature birth [44]. However, inadequate nutrition in the context of severe maternal weight loss could also contribute to a higher risk of both SGA and preterm birth [45].

## Conclusions

Our study highlights the importance of postpartum weight management, but also identifies opportunities for future research. There is a need to capture the typical weight change profiles of women in various BMI classes, to further elucidate risk groups. This will support further research into weight management strategies, eventually aiming to implement evidence-based weight control interventions to benefit maternal and offspring health. It is particularly important to elucidate strategies for postpartum weight loss in normal weight women, as this group might not be the focus of current research and interventions, yet may be at highest risk of adverse outcomes from interpregnancy weight gain.

In conclusion, we show that interpregnancy weight gain impacts on the risk of developing perinatal complications in a subsequent pregnancy and it is possible that BMI at the index pregnancy modifies these associations. These findings highlight the need to encourage women to return to their pre-pregnancy weight before conceiving again in an effort to reduce the risk of perinatal complications. Future work should focus on defining the most effective strategies to achieve this outcome.

## Supplementary information

**Additional file 1: Figure S1.** Forest plot from random effects meta-analysis showing the crude odds ratios for the association between interpregnancy weight loss and the risk for perinatal outcomes of interest. Black, solid dots represent studies with reference group of interpregnancy weight change between − 1 and + 1 BMI unit and are therefore included in the meta-analyses. White, open dots represent studies not using a reference group of interpregnancy weight change between 1-unit weight loss and 1-unit weight gain and are visually displayed but not included in the meta-analysis. cOR, crude odds ratio; CI, confidence interval.

**Additional file 2: Figure S2.** Forest plot from random effects meta-analysis showing the crude odds ratios for the association between interpregnancy weight gain and the risk for perinatal outcomes of interest. Black, solid dots represent studies with reference group of interpregnancy weight change between − 1 and + 1 BMI unit and are therefore included in the meta-analyses. White, open dots represent studies not using a reference group of interpregnancy weight change between 1-unit weight loss and 1-unit weight gain and are visually displayed but not included in the meta-analysis. cOR, crude odds ratio; CI, confidence interval.

**Additional file 3: Table S1.** Search string.

**Additional file 4: Table S2.** Assessment of study quality through the Newcastle–Ottawa scale.

## Data Availability

All data generated or analysed during this study are included in this published article and its additional file.

## Abbreviations

BMI: Body mass index
CI: Confidence interval
CS: Caesarean section
GDM: Gestational diabetes mellitus
PE: Preeclampsia
PIH: Pregnancy induced hypertension
NOS: Newcastle-Ottawa scale
aOR: adjusted odds ratio
cOR: crude odds ratio
PRISMA: Preferred reporting items for systematic reviews and meta-analysis PTB Preterm birth
LGA: Large-for-gestational age
SGA: Small-for-gestational age
