# Supplementary figures and images for: Effect of interpregnancy weight change on perinatal outcomes: systematic review and meta-analysis
Source: BMC Pregnancy Childbirth. 2019 Oct 28;19:386. doi: 10.1186/s12884-019-2566-2 (PMC6819632; doi:10.1186/s12884-019-2566-2)

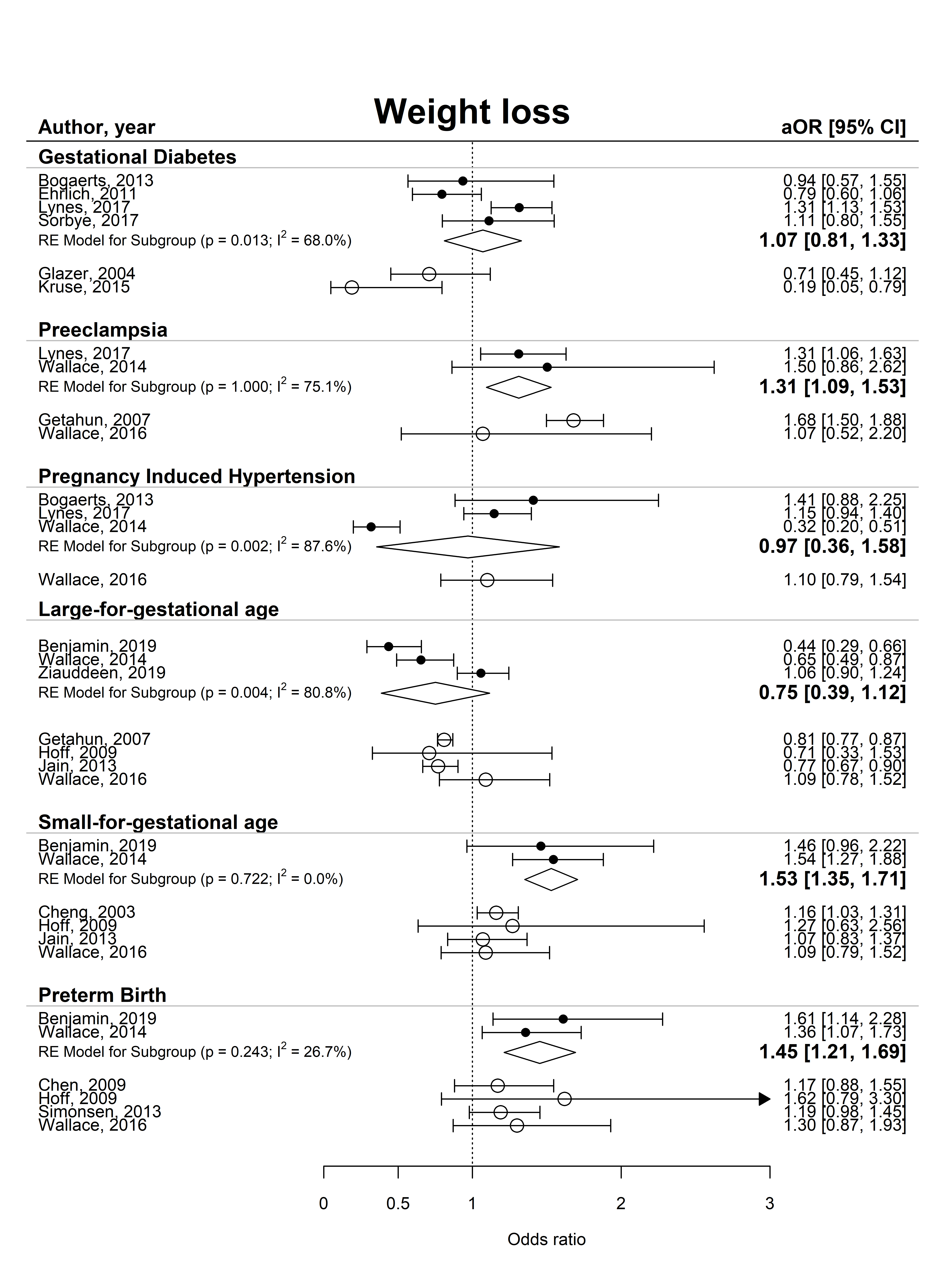

Supplement: Supplementary file 1 — Additional file 1: Figure S1. Forest plot from random effects meta-analysis showing the crude odds ratios for the association between interpregnancy weight loss and the risk for perinatal outcomes of interest. Black, solid dots represent studies with reference group of interpregnancy weight change between − 1 and + 1 BMI unit and are therefore included in the meta-analyses. White, open dots represent studies not using a reference group of interpregnancy weight change between 1-unit weight loss and 1-unit weight gain and are visually displayed but not included in the meta-analysis. cOR, crude odds ratio; CI, confidence interval. [file 12884_2019_2566_MOESM1_ESM.tiff]

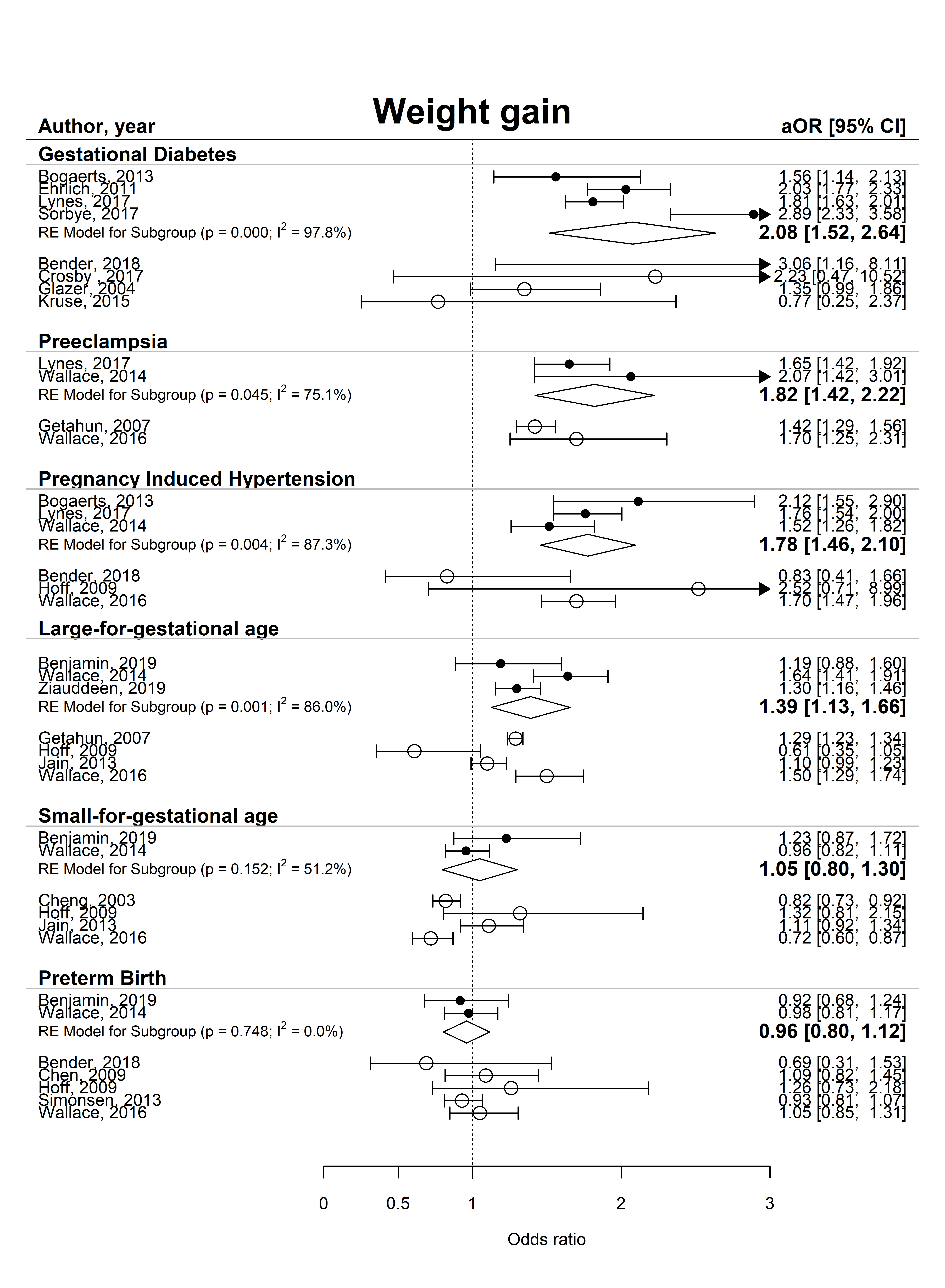

Supplement: Supplementary file 2 — Additional file 2: Figure S2. Forest plot from random effects meta-analysis showing the crude odds ratios for the association between interpregnancy weight gain and the risk for perinatal outcomes of interest. Black, solid dots represent studies with reference group of interpregnancy weight change between − 1 and + 1 BMI unit and are therefore included in the meta-analyses. White, open dots represent studies not using a reference group of interpregnancy weight change between 1-unit weight loss and 1-unit weight gain and are visually displayed but not included in the meta-analysis. cOR, crude odds ratio; CI, confidence interval. [file 12884_2019_2566_MOESM2_ESM.tiff]
