# Supplementary material for: Effect of interpregnancy weight change on perinatal outcomes: systematic review and meta-analysis
Source: BMC Pregnancy Childbirth. 2019 Oct 28;19:386. doi: 10.1186/s12884-019-2566-2 (PMC6819632; doi:10.1186/s12884-019-2566-2)
Supplement: Supplementary file 3 — Additional file 3: Table S1. Search string. [file 12884_2019_2566_MOESM3_ESM.docx]

Supplementary Table 1: Search string^[[1]](#footnote-1)^

| Interpregnancy OR Inter-pregnancy OR Inter pregnancy OR Between pregnancy OR Between pregnancies | | | | | | | | | | |
| --- | --- | --- | --- | --- | --- | --- | --- | --- | --- | --- |
| AND | | | | | | | | | | |
| Weight change OR BMI OR Body Mass Index | | | | | | | | | | |
| AND | | | | | | | | | | |
| Small for gestational age,  SGA,  fetal growth restriction,  foetal growth restriction,  fetal growth retardation,  foetal growth retardation,  FGR,  Intrauterine growth restriction,  Intrauterine growth retardation,  IUGR | OR | Large for gestational age,  LGA | OR | Preterm birth,  Pre-term birth,  Premature birth,  Pre-mature birth,  Preterm labour,  Pre-term labour,  Preterm labor,  Pre-term labor,  Prematurity | OR | Gestational diabetes,  Gestational diabetes mellitus,  Pregnancy induced diabetes,  Pregnancy-induced diabetes,  GDM | OR | gestational hypertension,  pregnancy-induced hypertension,  pregnancy induced hypertension, | OR | Pre-eclampsia,  pre-eclampsia |

1. Search string as used in Pubmed, OVID Embase and Cochrane Central. Search executed on 24^th^ July 2019. [↑](#footnote-ref-1)
