# Supplementary material for: Effect of interpregnancy weight change on perinatal outcomes: systematic review and meta-analysis
Source: BMC Pregnancy Childbirth. 2019 Oct 28;19:386. doi: 10.1186/s12884-019-2566-2 (PMC6819632; doi:10.1186/s12884-019-2566-2)
Supplement: Supplementary file 4 — Additional file 4: Table S2. Assessment of study quality through the Newcastle–Ottawa scale. [file 12884_2019_2566_MOESM4_ESM.docx]

|  | Bogaerts 2013 | Bender 2018 | Benjamin 2019 | Chen 2009 | Cheng 2003 * | Crosby 2017 | Erhlich 2011 | Getahun 2007 | Getahun 2007 | Glazer 2004 | Hoff 2009 | Jain 2013 | Knight-Agarwal 2016 | Kruse 2015 | Lynes 2017 | McBain 2016 | Pole 1999 | Simonsen 2013 | Sorbye 2017 | Villamor 2006 | Wallace 2014 | Wallace 2016 | Ziauddeen 2019 |
| --- | --- | --- | --- | --- | --- | --- | --- | --- | --- | --- | --- | --- | --- | --- | --- | --- | --- | --- | --- | --- | --- | --- | --- |
| **Selection** | | | | | | | | | | | | | | | | | | | | | | | |
| 1 | * | * | * | * | ** | - | * | * | * | * | * | * | * | - | * | * | * | * | * | * | * | * | * |
| 2 | * | * | * | * | - | * | * | * | * | * | * | * | * | * | * | * | * | * | * | * | * | * | * |
| 3 | - | * | - | - | * | * | * | - | - | - | - | - | * | - | - | * | - | - | - | * | * | * | * |
| 4 | - | - | - | * | * | - | - | * | - | * | - | - | - | * | - | - | - | * | * | - | * | * | * |
| **Comparability** | | | | | | | | | | | | | | | | | | | | | | | |
| 5 | ** | -- | -- | *- | *- | -- | ** | *- | *- | -- | *- | *- | ** | *- | -* | *- | -- | -- | *- | ** | ** | *- | ** |
| **Outcome** | | | | | | | | | | | | | | | | | | | | | | | |
| 6 | - | * | - | ** | - | - | ** | ** | ** | - | * | ** | - | ** | * | * | * | ** | - | - | ** | ** | ** |
| 7 | * | * | * | * | - | * | * | * | * | * | * | * | - | * | * | - | - | * | * | - | * | * | * |
| **Total (out of 9)** | | | | | | | | | | | | | | | | | | | | | | | |
|  | 5 | 5 | 3 | 7 | 5 | 3 | 8 | 7 | 6 | 4 | 5 | 6 | 5 | 6 | 5 | 5 | 3 | 6 | 5 | 5 | 9 | 8 | 9 |

Supplementary table 2: Assessment of study quality through the Newcastle–Ottawa Scale
